# Plasma Instead of Serum Avoids Critical Confounding of Clinical Metabolomics Studies by Platelets

**DOI:** 10.1021/acs.jproteome.3c00761

**Published:** 2024-03-23

**Authors:** Gerhard Hagn, Samuel M. Meier-Menches, Günter Plessl-Walder, Gaurav Mitra, Thomas Mohr, Karin Preindl, Andreas Schlatter, Doreen Schmidl, Christopher Gerner, Gerhard Garhöfer, Andrea Bileck

**Affiliations:** † Department of Analytical Chemistry, Faculty of Chemistry, University of Vienna, Waehringer Straße 38, 1090 Vienna, Austria; ‡ Vienna Doctoral School in Chemistry (DoSChem), University of Vienna, Waehringer Str. 42, 1090 Vienna, Austria; § Joint Metabolome Facility, University and Medical University of Vienna, WaehringerStraße 38, 1090 Vienna, Austria; ∥ Department of Laboratory Medicine, Medical University of Vienna, Waehringer Gürtel 18-20, 1090 Vienna, Austria; ⊥ Department of Clinical Pharmacology, Medical University of Vienna, 1090 Vienna, Austria

**Keywords:** acetylsalicylic acid, clinical metabolomics, confounders, serum, drug effects, lipid mediators, metabolomics, omega-3 fatty acids, plasma, platelets

## Abstract

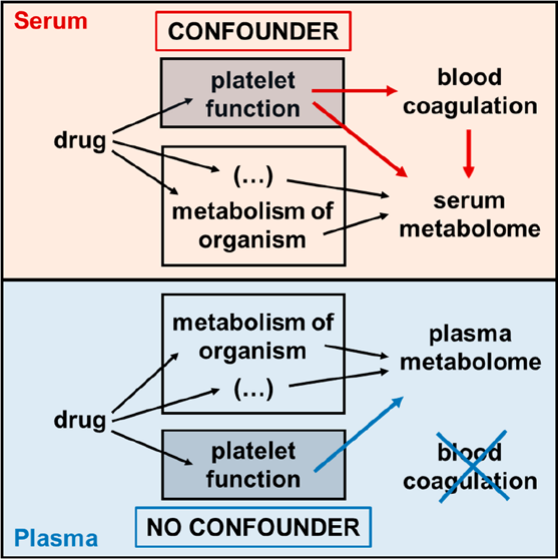

Metabolomics is an emerging and powerful bioanalytical method supporting clinical investigations. Serum and plasma are commonly used without rational prioritization. Serum is collected after blood coagulation, a complex biochemical process involving active platelet metabolism. This may affect the metabolome and increase the variance, as platelet counts and function may vary substantially in individuals. A multiomics approach systematically investigating the suitability of serum and plasma for clinical studies demonstrated that metabolites correlated well (*n* = 461, *R*^2^ = 0.991), whereas lipid mediators (*n* = 83, *R*^2^ = 0.906) and proteins (*n* = 322, *R*^2^ = 0.860) differed substantially between specimen. Independently, analysis of platelet releasates identified most biomolecules significantly enriched in serum compared to plasma. A prospective, randomized, controlled parallel group metabolomics trial with acetylsalicylic acid administered for 7 days demonstrated that the apparent drug effects significantly differ depending on the analyzed specimen. Only serum analyses of healthy individuals suggested a significant downregulation of TXB2 and 12-HETE, which were specifically formed during coagulation *in vitro*. Plasma analyses reliably identified acetylsalicylic acid effects on metabolites and lipids occurring *in vivo* such as an increase in serotonin, 15-deoxy-PGJ2 and sphingosine-1-phosphate and a decrease in polyunsaturated fatty acids. The present data suggest that plasma should be preferred above serum for clinical metabolomics studies as the serum metabolome may be substantially confounded by platelets.

## Introduction

Metabolomics represents a contemporary postgenomic analysis method for small molecules, comprising building blocks for biosynthesis, fuel for energy production along with the corresponding waste products as well as catalytically active metabolites and signaling molecules.^1^ In contrast to other biomolecules, metabolites may be formed and degraded by a variety of different and independent mechanisms, rendering data interpretation more difficult and calling for supportive machine learning algorithms.^2^ However, metabolomics data may improve the diagnosis of diseases, help to better understand disease mechanisms, and represent an important tool to practice precision medicine supporting individualized drug treatments and monitoring therapeutic outcomes.^3^

Clinical metabolomics is typically performed using serum or plasma as sample matrices. The preparation of serum implies blood coagulation before centrifuging off the particular part of the full blood. In contrast, blood coagulation is inhibited on purpose by adding an anticoagulant to obtain plasma. As blood coagulation implies the functional activation of platelets, some differences in the metabolome composition of serum and plasma may be expected. Indeed, several studies investigated and reported such differences between the metabolite composition of serum and plasma.^4−8^ Interindividual variations, age and sex-related differences were described to be rather similar between serum and plasma,^9^ and a recent study provided practically useful information regarding serum and plasma protein and metabolite stability during sample preparation.^10^ However, these studies did not yet result in a mandatory recommendation of what to prefer, as all of them only investigated serum and plasma without performing comparative interventional studies. Here we also considered that such interventional studies may be required to decisively conclude whether serum or plasma was to be preferred for clinical investigations. With an increasing relevance of metabolomics for precision medicine, it is high time to decide for either plasma or serum based on a clear rationale resulting in the improved standardization of clinical metabolomics.

Thus, we have performed systematic studies based on the assumption that platelets may affect the serum metabolome due to blood coagulation occurring in the course of sample preparation. Detectable differences between serum and plasma were confirmed to be caused by platelet activation during blood coagulation. Indeed, almost all molecules significantly up-regulated in serum when compared to plasma were found to be contained in platelet releasates.

This observation raised important questions regarding the clinical investigation of drug effects by serum metabolomics. We and others have observed individual variations regarding functional responses to nutritional interventions^11^ or defined challenges such as cytokine formation upon inflammatory stimulation.^12^ It is well established that platelet counts may vary substantially between individuals and also within individuals at different time points.^13^ In addition, it is evident that all kinds of drugs interfering with inflammatory processes, arteriosclerosis, or blood coagulation will affect platelet function and metabolism. This points to an unavoidable confounding of serum metabolomics data caused by the unobserved variation of platelet counts and functions.

In order to clarify these potential issues, we have conducted a prospective, randomized, controlled parallel group interventional study to assess potential differences between the serum and plasma metabolome after a seven day administration of two widely used and well understood agents, acetylsalicylic acid and omega-3 fatty acids. Acetylsalicylic acid was chosen because it is an antiphlogistic drug inhibiting enzymatic cyclooxygenase activities, thus affecting platelet activation.^14,15^ The implications of long-term low-dose treatment of vascular diseases and colon cancer with acetylsalicylic acid are still a matter of debate.^16^ Omega-3 fatty acid supplements were chosen as they are precursors of lipid mediators, and to the best of our knowledge, this should have no direct effect on platelet functions.

The commercial validated Biocrates MxP Quant 500 kit^17,18^ was used for serum and plasma metabolomics. In parallel, proteome profiling was performed to support data interpretation. Furthermore, fatty acids and lipid mediators were analyzed with an eicosadomics assay established in our laboratory, as platelets are actively forming these special class of metabolites known to be involved in many diseases.^17,19^ The prospective design under tightly controlled conditions was chosen to make potential differences in the metabolomics outcomes, dependent on the choice for serum or plasma obtained from the same individuals, fully transparent and understandable.

## Experimental Section

### Study Design

Subjects were recruited by the Department of Clinical Pharmacology at the Medical University of Vienna. The study protocol was approved by the Ethics Committee of the Medical University of Vienna (EC No. 2250/2020) and the Austrian competent authorities. The study was conducted in accordance with the Declaration of Helsinki and Good Clinical Practice (GCP) guidelines of the European Union. Written informed consent was obtained from all of the study participants prior to study entry. The study design was a randomized, controlled, parallel group study. For each of the following three study cohorts, i.e., (a) comparison of serum and plasma, (b) treatment with acetylsalicylic acid and (c) Omega-3 treatment, 6 healthy individuals were enrolled (Supplementary Table S1). Subjects were included only if no abnormalities were found at the screening visit. Exclusion criteria compiled symptoms of a clinically relevant illness in the 3 weeks before the first study day, a severe medical condition, or the usage of any concomitant medication (except contraceptives) or dietary supplements within 3 weeks before the first study day.

Subjects were randomized to receive either acetylsalicylic acid or Omega-3 capsules for 7 days. One study cohort was instructed to take 500 mg of acetylsalicylic acid (Aspirin 500 mg of acetylsalicylic acid, cellulose powder, maize starch) per day in the evening, whereas the second study cohort was instructed to take two Omega-3 complex 870 mg capsules (Dr. Böhm Omega-3 capsules, 1017 mg cold water fish oil equivalent to 870 mg Omega-3, consisting of 420 mg EPA, 330 mg DHA, 5 μg Vitamin D equivalent to 200 IU, 6 mg Vitamin E, 30 mg Coenzyme Q10) per day in the evening.

### Sample Collection

Blood samples were obtained at baseline and after 7 days of intake of the study medication. On both study days two blood samples using 6 mL of K3EDTA and serum collection tubes (both Vacuette, Greiner Bio-One GmbH, Kremsmünster, Austria) were obtained from each subject. EDTA-anticoagulated tubes were carefully inverted two times after blood draw and centrifuged immediately at room temperature (2000*g*) for 10 min. In contrast, filled serum tubes were carefully inverted after blood draw and placed to sit upright for 15 to 30 min to allow clot formation. Then, the tubes were centrifuged at room temperature at 2000*g* for 10 min. Directly after centrifugation, 500 μL of plasma or serum, respectively, were transferred into prelabeled Eppendorf safe-lock tubes and stored at −80 °C until analysis.

### Serum and Plasma Proteomics

Samples were diluted 1:20 in lysis buffer (8 M urea, 50 mM triethylammonium bicarbonate (TEAB), 5% sodium dodecyl sulfate (SDS)), heated at 95 °C for 5 min prior to determination of protein concentration using a BCA assay. Enzymatic digest of 20 μg of protein sample was achieved by applying the ProtiFi S-trap technology.^20^ Briefly, solubilized protein samples were reduced and carbamidomethylated before loading them onto the S-trap mini cartridges. Afterward, samples were washed and digested using Trypsin/Lys-C Mix at 37 °C for 2 h. Peptides were eluted, dried, and stored at −20 °C until liquid chromatography–tandem mass spectrometry (LC-MS/MS) analyses.

LC-MS/MS analysis was performed using a Dionex Ultimate3000 nanoLC-system (Thermo Fisher Scientific) coupled to the timsTOF Pro mass spectrometer (Bruker) equipped with a captive spray ion source as described previously.^17,18,21^ Briefly, dried peptide samples were reconstituted in 5 μL of 30% formic acid (FA) containing standard peptides and diluted with 40 μL of loading solvent (97.9% H_2_O, 2% acetonitrile (ACN), 0.05% trifluoroacetic acid). Thereof, 1 μL was injected for LC-MS/MS analysis. Peptide separation was achieved on an analytical column [25 cm × 75 μm, 1.6 μm C18 Aurora Series emitter column (IonOpticks)] by applying a flow rate of 300 nL/min and using a gradient of 7% to 40% mobile phase B (79.9% ACN, 20% H_2_O, 0.1% FA) mixed with mobile phase A (99.9% H_2_O, 0.1% FA) over 43 min. Including column washing and equilibration phases, this results in a total run time of 85 min. The mass spectrometer was operated in the Parallel Accumulation-Serial Fragmentation mode.

Data analysis including protein identification and label-free quantification (LFQ) was accomplished using MaxQuant 1.6.17.0.^22^ Raw data were searched against the SwissProt database “homo sapiens” (version 141219 with 20380 entries) including an allowed peptide tolerance of 20 ppm, a maximum of two missed cleavages, carbamidomethylation on cysteins as fixed modification, as well as methionine oxidation and N-terminal protein acetylation as variable modification. A minimum of one unique peptide per protein was set as a search criterion for positive identifications. The “match between runs” option was applied. For all peptide and protein identification, a false discovery rate (FDR) ≤ 0.01 was set. Using Perseus 1.6.14.0^23^ identified proteins were filtered for reversed sequences and common contaminants. LFQ intensities were transformed (log_2_(*x*)), and proteins were additionally filtered for their number of independent identifications (proteins identified in 70% of samples in at least one group). Missing values were replaced from a normal distribution, and a principal component analysis (PCA) was performed.

### Serum and Plasma Eicosadomics

LC-MS/MS analysis of lipid mediators was performed as described previously.^17,18,24^ Briefly, 400 μL was mixed with 1.6 mL of cold ethanol (EtOH) including an internal eicosanoid standard mixture for protein precipitation and stored at −20 °C overnight. The internal standard mix consists of 12*S*-hydroxyeicosatetraenoic acid (HETE)-*d*_8_, 15S-HETE-*d*_8_, 20-HETE-*d*_6_, 5-oxo-eicosatetraenoic acid (ETE)-*d*_7_, prostaglandin E2 (PGE2)-*d*_4_ and 11,12-dihydroxy-5*Z*,8*Z*,14*Z*-eicosatrienoic acid (DiHETrE)-*d*_11_ (Cayman Chemical, Tallinn, Estonia). The exact concentrations of each internal standard can be found in Supplementary Table S2. Solid phase extraction (SPE) was performed using StrataX SPE columns (30 mg mL^–1^; Phenomenex, Torrance, CA, USA). Afterward, samples were dried and then reconstituted in 150 μL of reconstitution buffer (H_2_O:ACN:methanol (MeOH) + 0.2% FA–vol % 65:31.5:3.5).

LC-MS/MS analyses were performed using a Thermo Scientific Vanquish (UHPLC) system coupled to a Q Exactive HF Orbitrap high-resolution mass spectrometer (Thermo Fisher Scientific, Austria) equipped with an HESI source for negative ionization. Separation of analytes was achieved using a Kinetex C18-column (2.6 μm XB-C18 100 Å, LC Column 150 × 2.1 mm; Phenomenex) and applying a flow gradient. Twenty μL of each sample was injected and all samples were analyzed in technical duplicates. The MS scan range was set to 250–700 *m*/*z* with a resolution of 60,000 (at *m*/*z* 200) on the MS1 level. A Top 2 method was applied for fragmentation (HCD 24 normalized collision energy) as well as an inclusion list covering 33 *m*/*z* values specific for well-known eicosanoids and precursor molecules (Supplementary Table S3). The resulting fragments were analyzed on the MS2 level at a resolution of 15,000 (at *m*/*z* 200). Operating in negative ionization mode, a spray voltage of 3.5 kV and a capillary temperature of 253 °C were applied. Sheath gas was set to 46 and the auxiliary gas was set to 10 (arbitrary units).

For data analysis, analytes were compared to an in-house established database on the MS1 level based on exact mass and retention time (degree of identification shown in Supplementary Table S4) by using the TraceFinder software (version 4.1). Subsequently, MS/MS fragmentation spectra were manually compared to reference spectra of in-house measured, commercially available standards or to reference spectra from the Lipid Maps depository library in July 2018.^25^ Relative quantification of the identified analytes was then performed on the MS1 level by using the TraceFinder software (version 4.1). Resulting data were loaded into the R software package environment (version 4.2.0).^26^ Peak areas were log_2_-transformed and normalized to the internal standards. For normalization, the mean log_2_-transformed peak area of the internal standards was subtracted from the log_2_-transformed analyte peak areas to correct for variances arising from sample extraction and LC-MS/MS analysis. Log_2_-transformed normalized areas were increased by adding (*x* + 20) to obtain a similar value distribution compared to label-free quantification in proteomics and, thus, enable missing value imputation. Missing values were imputed using the minProb function of the imputeLCMD package (version 2.1).^27^ Principal component analysis was performed using Perseus 1.6.14.0.^23^

### Serum and Plasma Metabolomics

Targeted metabolomics experiments were conducted by applying the MxP Quant 500 Kit (Biocrates Life Sciences AG, Innsbruck, Austria) as described previously.^17,18^ Therefore, 10 μL of sample was used and the kit was performed according to the manufacturer’s instructions. Measurements were carried out using LC-MS/MS and flow injection (FIA)-MS/MS analyses on a Sciex 6500+ series mass spectrometer coupled to an ExionLC AD chromatography system (AB Sciex, Framingham, MA, USA), utilizing Analyst 1.7.1 software with hotfix 1 (also AB SCIEX). All required standards, quality controls, and eluents were included in the kit, as well as the chromatographic column for the LC-MS/MS analysis part. Preparation of the measurement worklist and data validation and evaluation were performed with the software supplied with the kit (MetIDQ-Oxygen-DB110-3005, Biocrates Life Sciences). Out of the 630 included analytes, a total of 461 metabolites showed signal intensities within the quantification window and were further evaluated. Analytical figures of merit are shown in Supplementary Table S5. Principal component analysis was performed using Perseus 1.6.14.0.^23^

### Platelet Isolation, Activation, and LC-MS/MS Analyses

Whole blood of six healthy donors (three male and three female) in the age range of 26 to 51 years were collected in biological duplicates with 1 week in between the donations, resulting in 12 biological samples. Each donor gave written consent, and the study was approved by the ethics committee of the Medical University of Vienna in accordance with the Declaration of Helsinki (EC 1430/2018). No medical substances interfering with the normal physiology of platelets such as aspirin, paracetamol, or ibuprofen were taken by the donors 48 h prior to blood donation. Two CPDA (citrate-phosphate-dextrose-adenine)-S-Monovette tubes (Sarstedt) of venous blood were collected per donor and donation. To isolate platelet rich plasma (PRP), the tubes were centrifuged for 20 min at 100*g* with acceleration and deceleration set to 4.

To purify platelets, size exclusion chromatography using 2% B agarose beads (50–150 μm; abtbeads.es) was performed. Therefore, columns were equipped with a cotton frit and 20 mL of reconstituted agarose bead solution diluted 1:2 in RPMI medium (1× with l-glutamine; Gibco, Thermo Fisher Scientific, Austria). Columns were washed with 2 mL of RPMI medium before 1 mL of PRP was carefully pipetted to the column and topped with RPMI. Two columns per donor and donation were used. The fractions containing purified platelets of each donor were pooled in order to obtain a homogeneous sample and afterward divided in two aliquots, one for platelet activation and one serving as control. To achieve platelet activation, ionomycin calcium salt (Sigma-Aldrich) was added to one aliquot to a final concentration of 1 μM. As a 100 μM ionomycin stock solution containing 0.77% DMSO was used, the control platelets were treated with a DMSO vehicle control accordingly. All samples were incubated for 15 min at room temperature before centrifugation at 2000*g* for 5 min. The supernatant was transferred into new tubes, and protein precipitation was performed by adding ice cold ethanol (LC-MS grade) in a ratio of 1:5. Additionally, 5 μL of an internal standard mixture of 12S-HETE-*d*_8_, 15*S*-HETE-*d*_8_, 5-oxo-eicosatetraenoic acid (ETE)-*d*_7_, 11,12-dihydroxy-5*Z*,8*Z*,14*Z*-eicosatrienoic acid (DiHETrE)-*d*_11_, PGE2-d4 and 20-HETE-*d*_6_ (Cayman Europe, Tallinn, Estonia) were added to each sample. The exact concentrations of each internal standard can be found in Supplementary Table S2. Samples were then stored at −20 °C. After overnight precipitation, samples were centrifuged for 30 min at 4536*g* at +4 °C. The supernatant was then transferred into new 15 mL Falcon tubes and submitted to the lipid extraction workflow in order to enable LC-MS/MS analysis of fatty acids and lipid mediators as described above for serum and plasma samples. The remaining pellets representing secreted proteins were dried in an exsiccator and submitted to the proteomics workflow, as described above, for serum and plasma samples.

### Statistical Analysis and Graphical Visualization

For statistical analyses log_2_ transformed expression values were loaded into R,^28^ and fitted to a linear model using LIMMA^29^ with subjectID as pairing variable where appropriate. *P*-values were adjusted for multiple testing according to Benjamini–Hochberg.^30^ Volcano plots representing log_2_ fold-changes on the *x*-axis and -log adjusted *p*-values on the *y*-axis were generated using GraphPad Prism Version 6.07 (2015). Molecules displaying a fold-change of ≥ or ≤2 and an adjusted *p*-value of ≤0.05 were considered as statistically significant. Pearson correlation analyses between serum and plasma were performed separately for proteins, fatty acid, and lipid mediators as well as metabolites using GraphPad Prism Version 6.07 (2015). The effect of acetylsalicylic acid on a subset of 136 triglycerides (TGs) was shown by means of a linear regression and Spearman correlation analyses, again using GraphPad Prism Version 6.07 (2015). Therefore, fold-changes of C:16 and C:18 TGs in serum and plasma, respectively, before and after 7 days of acetylsalicylic acid intake were correlated to the sum of C atoms of the two remaining fatty acids. Heatmap visualizing fold-changes of C:16 and C:18 TGs in serum and plasma, respectively, before and after 7 days of acetylsalicylic acid intake was generated using Microsoft Excel.

### Data Sharing Statement

All proteomics data was submitted to the ProteomeXchange Consortium (http://proteomecentral.proteomexchange.org) and is available in the PRIDE partner repository^31^ with the data set identifier PXD041781 and PXD041785.

Metabolomics data as well as data derived from the lipid mediator analysis are available at the NIH Common Fund’s National Metabolomics Data Repository (NMDR) Web site, the Metabolomics Workbench, https://www.metabolomicsworkbench.org,^32^ where they have been assigned to following studies:

Oxylipins of serum and plasma samples: Study ID ST003050, directly accessible via its Project DOI: 10.21228/M88147.

Oxylipins of platelet samples: Study ID ST003049, directly accessible via its Project DOI: 10.21228/M88147.

Plasma and serum metabolomics: Study ID ST003069, directly accessible via its Project DOI: 10.21228/M88147.

## Results and Discussion

### Molecular Characterization of Serum and Plasma Samples

**Figure 1:**
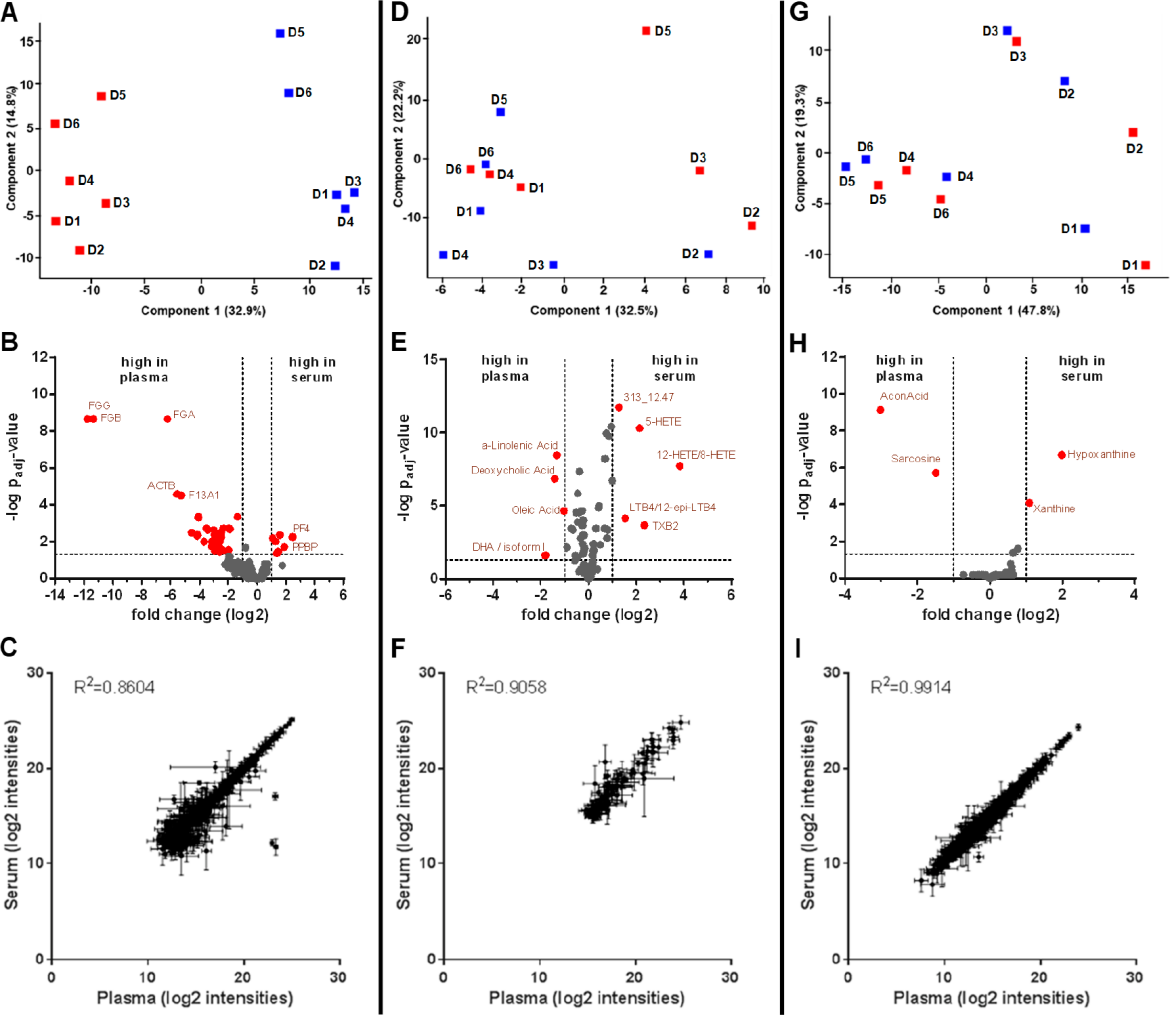
Molecular characterization of serum and plasma samples. Principal component analyses (red = serum samples; blue = plasma samples; D1–D6 = Donor 1–Donor 6), volcano plots showing significant differences of molecules between serum and plasma (positive fold-changes mean higher abundance in serum compared to plasma), and correlation analyses are shown for (A–C) proteins, (D–F) fatty acids and lipid mediators, and (G–I) metabolites. For statistical analyses, data were fitted to a linear model using LIMMA with subjectID as a pairing variable, and *p*-values were adjusted for multiple testing according to Benjamini–Hochberg. Molecules displaying a fold-change of ≥ or ≤2 and an adjusted *p*-value of ≤0.05 were considered as statistically significant and marked in red in the corresponding volcano plot.

In order to investigate the molecular composition of plasma in comparison to the serum of untreated healthy donors, venous blood was drawn from six individuals. Serum and plasma samples were obtained from each blood donation. Proteome profiling based on label-free shotgun analysis identified 322 proteins in total and demonstrated the almost complete loss of the fibrinogen subunits in serum, as expected due to blood coagulation. Seven proteins were apparently upregulated in serum when compared to plasma (Figure 1B, Supplementary Table S6). Principle component analysis (PCA) separated serum and plasma samples (Figure 1A). The correlation coefficient of proteins detected in serum and plasma was found to be *R*^2^ = 0.860 (Figure 1C). The eicosadomics assay detected 83 different molecules reliably and reproducibly in at least one group, as detailed in Supplementary Table S6. Among those were 26 oxylipins, 22 lysophosphatidylcholins, 17 fatty acids, sphingosine-1-phosphate and 4 bile acids. Thirteen Molecules were identified as oxylipins based on the sum formula, the isotopic pattern, and the molecular fragment ions. However, due to high structural ambiguity of oxylipin isobars we have not yet assigned unambiguous structures to these molecules, which have been designated here (Supplementary Table S6) as “molecular mass_chromatographic retention time”. The double bonds of polyunsaturated fatty acids such as DHA are typically all cis-configured, but may contain trans-configured double bonds distinguished by chromatography as described previously,^33^ designated accordingly as “Isoform I”, “Isoform II” or else. A comparison between serum and plasma identified 9 significantly different molecules (Figure 1E, Supplementary Table S6). The PCA hardly separated serum and plasma samples (Figure 1D) and the correlation coefficient was found to be *R*^2^ = 0.906 (Figure 1F). The metabolomics assay performed using the Biocrates MxP Quant 500 kit determined a total of 461 molecules and identified only 4 metabolites with significant concentration differences between serum and plasma (Figure 1H). Here, the PCA did not separate serum and plasma samples (Figure 1G) and the correlation coefficient was found as high as *R*^2^ = 0.991 (Figure 1I, Supplementary Table S6). This data suggested only minor differences in the metabolome as determined from serum and plasma isolated from healthy donors.

### Main Differences between Serum and Plasma Are a Consequence of Platelet Activation

**Figure 2:**
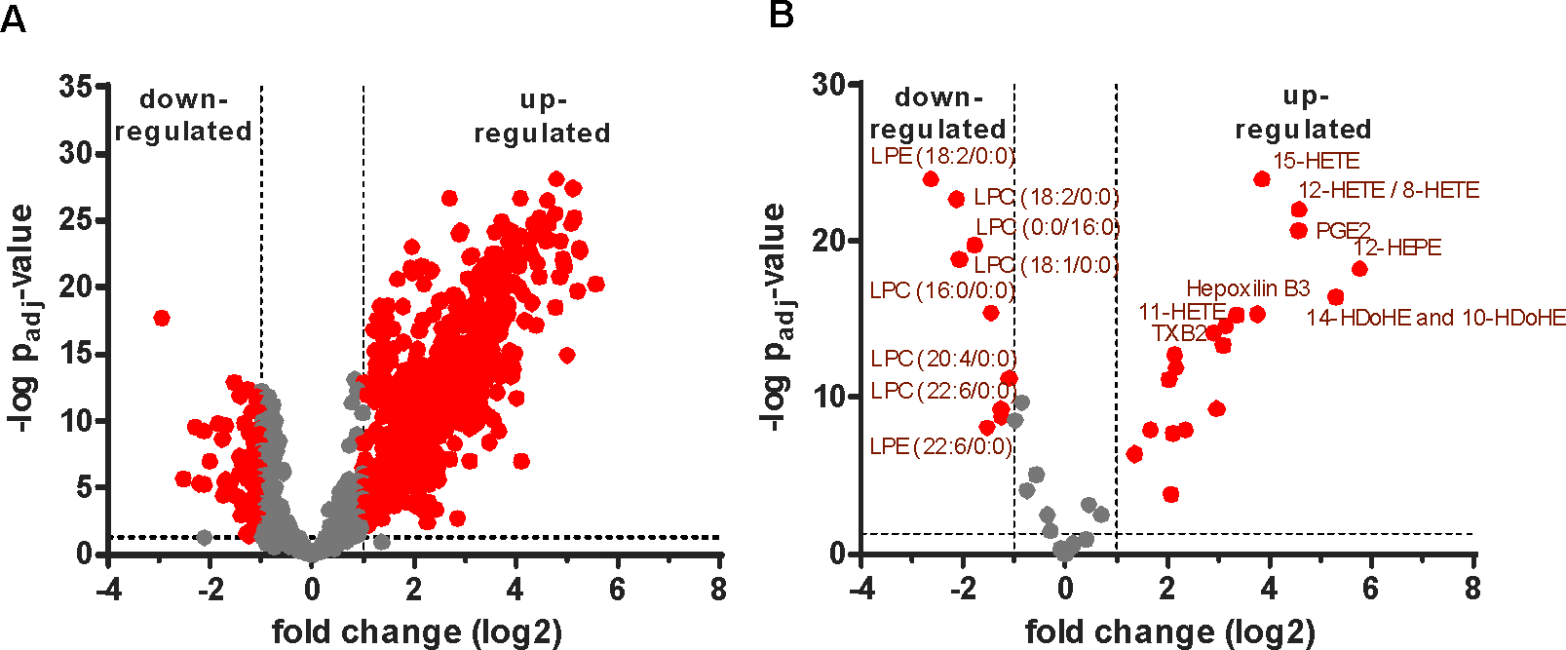
Molecular characterization of platelet releasates upon activation. Volcano plots showing significant regulations of (A) proteins and (B) lipid mediators upon platelet activation. *P*-values were calculated based on a linear model using LIMMA with subjectID as pairing variable and adjusted for multiple testing according to Benjamini–Hochberg. Molecules displaying a fold-change of ≥ or ≤2 and an adjusted *p*-value of ≤0.05 were considered as statistically significant and marked in red.

A functional annotation of the proteins apparently upregulated in serum, when compared to plasma, using the DAVID Bioinformatics Resources^34,35^ suggested platelets as their potential origin (GOBP: platelet activation, Benjamini–Hochberg adjusted *p*-value = 3.5 × 10^–3^; GOCC: platelet alpha granule lumen, Benjamini–Hochberg adjusted *p*-value = 9.7 × 10^–9^). In order to investigate this hypothesis in more detail, platelets were isolated and activated *in vitro* by the addition of ionomycin. The following platelet aggregation was found to be accompanied by the significant deregulation of 786 proteins, as well as 28 fatty acids and lipid mediators in the platelet supernatant (Figure 2, Supplementary Table S7). Most of these findings are compatible with or reproduce the results of previous reports.^19,36−38^ As expected, all proteins except antibodies with increased abundance values in serum, when compared to those in plasma, were also found to be released from platelets upon aggregation. Similarly, most eicosanoids with high abundance values in serum were found to be released from platelets upon aggregation (Supplementary Table S8). These observations supported the hypothesis that platelet releasates, formed during blood coagulation, contributed directly to the molecular composition of serum.

### Metabolic Alterations in Response to Acetylsalicylic Acid Administration Apparently Differ between Plasma and Serum

**Figure 3:**
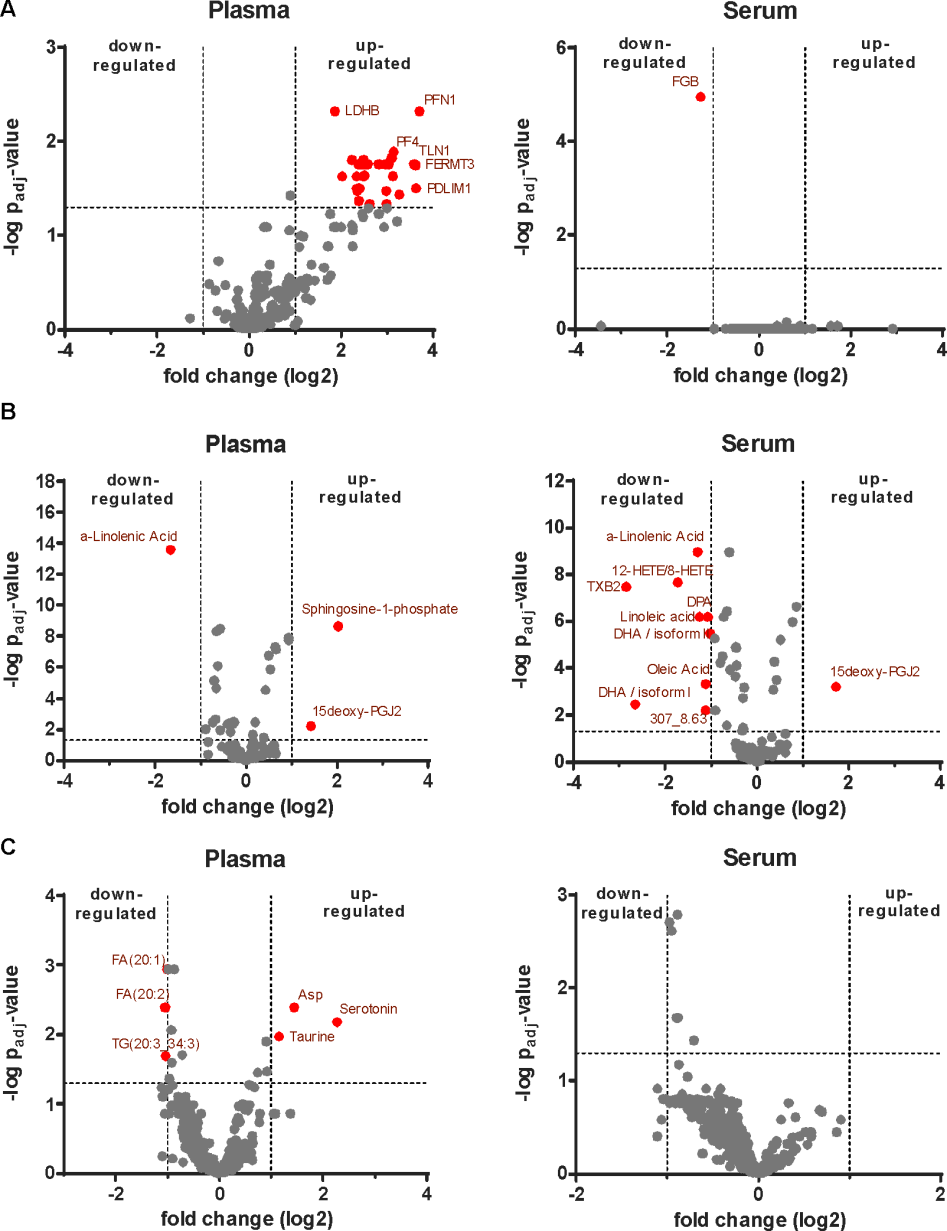
Effects of acetylsalicylic acid on the molecular profiles of serum and plasma, respectively. Volcano plots showing acetylsalicylic acid-induced effects on (A) proteins, (B) lipid mediators, and (C) metabolites in serum and plasma, respectively. *P*-values were calculated based on a linear model using LIMMA with subjectID as pairing variable and adjusted for multiple testing according to Benjamini–Hochberg. Molecules displaying a fold-change of ≥ or ≤2 and an adjusted *p*-value of ≤0.05 were considered as statistically significant and marked in red.

After comparing background levels of biomolecules between plasma and serum, we investigated molecular alterations in response to acetylsalicylic acid administration in a prospective, randomized, controlled parallel group trial. Proteome profiling of plasma revealed 32 proteins significantly upregulated upon drug exposure (Figure 3A). Thirty-one of those 32 proteins were found to be abundant in platelets. Acetylsalicylic acid has been described to affect platelet half-life,^39^ thus increasing the occurrence of platelet ghosts (dead platelets). As platelet ghosts fail to be centrifuged off during the isolation of plasma, they may cause this apparent up-regulation of platelet proteins (Supplementary Table S9). In contrast, only one protein, the fibrinogen beta chain, was found to be apparently deregulated in serum upon drug exposure (Figure 3A). Thus, serum and plasma analysis results regarding the effects of acetylsalicylic acid administration differed substantially.

The eicosadomics assay performed with the same samples demonstrated the significant downregulation of TXB2, 12-HETE and other fatty acids by acetylsalicylic acid in serum (Figure 3B, Supplementary Table S9). TXB2 and 12-HETE were among the most abundant eicosanoids released by platelets upon coagulation (Supplementary Table S8 and Supplementary Table S7). Thus, this data again pointed to the known mode of action of acetylsalicylic acid, inhibiting cyclooxygenases and platelet activation and thus the formation of these molecules, and reproduces previous findings.^15,40^ However, when the effects of acetylsalicylic acid administration in plasma were analyzed, some of these results obtained with serum were not reproduced. Two events were commonly observed in serum and plasma: the downregulation of a-linolenic acid, an omega-3 fatty acid, and the up-regulation of the anti-inflammatory 15-deoxy-PGJ2. Remarkably, in plasma sphingosine-1-phosphate was found up-regulated, whereas the eicosanoids TXB2 and 12-HETE were apparently not affected (Figure 3B, Supplementary Table S9). Sphingosine-1-phosphate is an important immune regulator described to be downregulated during inflammatory diseases such as atherosclerosis and sepsis.^41^ The acetylsalicylic acid induced up-regulation was not described up until now and may point to relevant adaptive responses.

**Figure 4:**
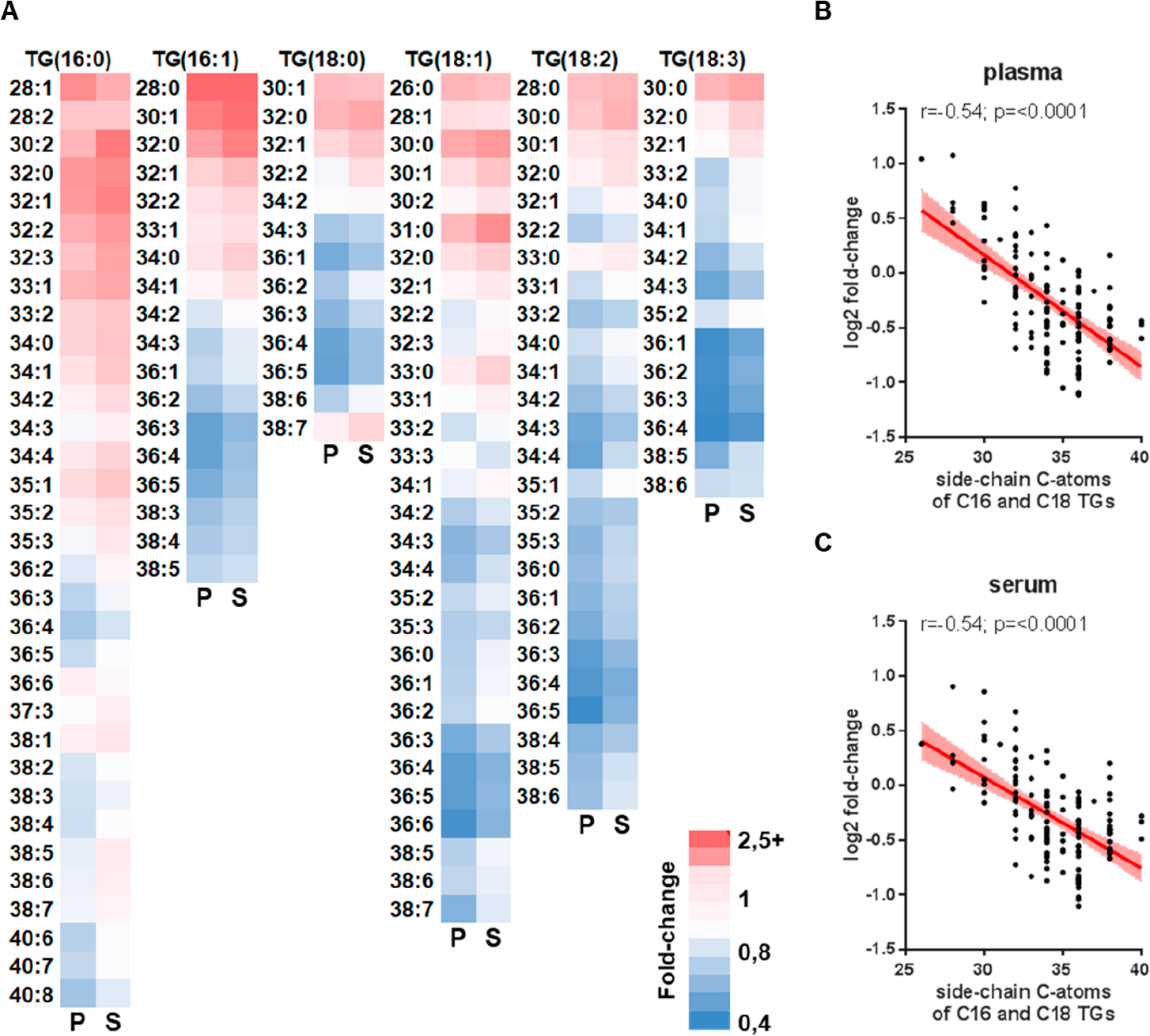
Effects of acetylsalicylic acid intake on the fatty acid composition of triglycerides. (A) Heatmap displaying the fold-changes of triglycerides (TGs) in plasma (P) and serum (S) when comparing samples before and after 7 days of acetylsalicylic acid intake. While fold-changes marked in red indicate upregulation of respective TGs upon acetylsalicylic acid intake, fold-changes marked in blue indicate higher levels of respective TGs before acetylsalicylic acid intake. Linear regression and Spearman correlation analyses between fold-changes of C:16 and C:18 TGs in (B) plasma and (C) serum, before and after 7 days of acetylsalicylic acid intake, and the sum of C atoms of the two remaining fatty acids are shown.

The administration of acetylsalicylic acid also induced alterations of some metabolites, as determined by the Biocrates MxP Quant 500 kit (Figure 3C). Here, triglycerides (TGs) are annotated by the sum of carbons and double bonds of the sn1 fatty acid, followed by the corresponding sum of the remaining two fatty acids. While only one single TG species, TG(20:3_34:3), was found significantly altered in plasma, Spearman correlation analyses demonstrated a significant loss of PUFAs in TGs accompanied by higher levels of saturated fatty acids when considering all detectable 136 TG species (Figure 4). This observation was independently reproduced in serum (Figure 4B, C), pointing to a consistent acetylsalicylic acid effect *in vivo*. A similar shift in the fatty acid composition of triacylglycerols has been previously described in case of metabolic syndrome.^42^ Remarkably, a systematic analysis of the biochemical effects of aspirin already described similar effects, actually downregulation of the intracellular content of PUFAs in *Saccharomyces cerevisiae*. This effect was attributed to aspirin-induced alterations in the expression of DCI1 and OLE2.^43^ The significance of this finding remains to be further explored, as such a mechanism—in line with the above-described deregulation of sphingosine-1-phosphate—might account for presently debated potential long-term side effects of aspirin such as dementia and cognitive decline.^44^

In addition, the metabolites serotonin, taurine, and asparagine were found significantly upregulated in plasma, but not in serum, upon acetylsalicylic acid administration. Increased serotonin will again relate to platelets and may account for the antidepressant effect of aspirin as described previously.^45^ Taurine and asparagine are both related to the stress response evidently taking place during inflammation and may thus rather indirectly relate to aspirin effects.

Considering important lipid mediators demonstrated that many metabolites observed to be altered when investigating the effects of acetylsalicylic acid in healthy subjects were confounded by the metabolism of platelets during blood coagulation. The serum data depicted in Figure 3B suggested that acetylsalicylic acid inhibited the formation of eicosanoids. Understanding the mechanistic steps involved in serum sample preparation tells us that this suggestion was misleading. In fact, acetylsalicylic acid inhibited eicosanoid formation taking place during blood coagulation, occurring after blood donation. The metabolome alterations observed in plasma may thus better report the actual drug effects taking place in the organism. Indeed, acetylsalicylic acid consumption seemed to have consequences beyond platelet inhibition. This was suggested by the drug-induced upregulation of serotonin, aspartic acid and taurine (Figure 3C), in addition to an alteration in the fatty acid composition of triacylglycerols (Figure 4). These observations are not novel, but in accordance with existing literature.^15,40,43,46^ However, the functional significance of these findings remains to be investigated in more detail.

### Metabolic Alterations in Response to Omega-3 Fatty Acids Administration Did Not Differ between Plasma and Serum

**Figure 5:**
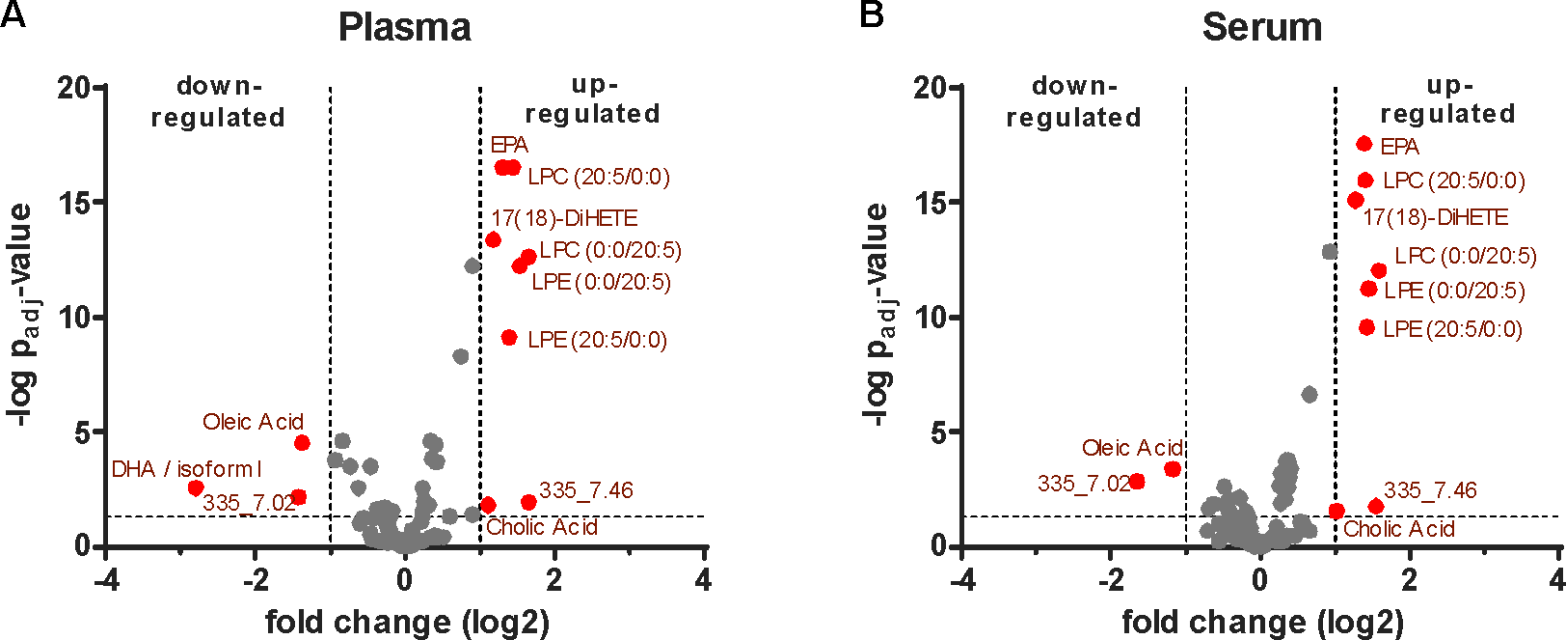
Effects of Omega-3 supplementation on fatty acids and lipid mediators in serum and plasma, respectively. Volcano plots showing Omega-3-induced effects on fatty acids and lipid mediators in (A) plasma and (B) serum. *P*-values were calculated based on a linear model using LIMMA with subjectID as pairing variable and adjusted for multiple testing according to Benjamini–Hochberg. Molecules displaying a fold-change of ≥ or ≤2 and an adjusted *p*-value of ≤0.05 were considered as statistically significant and marked in red.

Administration of omega-3 fatty acids, as performed in an independent prospective, randomized, controlled parallel group trial, resulted in the significant upregulation of 17(18)-dihydroxy-eicosatetraenoic acid (DiHETE), eicosapentaenoic acid (EPA), and EPA-containing lysophosphatidylcholine (LPC) and lysophosphatidylethanolamine (LPE) (Figure 5, Supplementary Table S10). Remarkably, these alterations were observed in both sample matrices, serum and plasma, in an almost identical fashion. It may be expected to detect an up-regulation of EPA, and anti-inflammatory EPA-related lipids such as 17(18)-DiHETE upon the administration of EPA, the main constituent of the administrated omega-3 fatty acids, as observed previously.^47^ However, the up-regulation of the EPA-containing lysolipids has not yet been described and points to a high turnover-rate of lipids in the human body.^48^ Similar effects with regard to DHA and DHA-related lipids, which were readily detectable in plasma,^17^ were not observed.

### Platelets Represent the Main Confounder Responsible for Differences between Serum and Plasma Metabolomics

**Figure 6:**
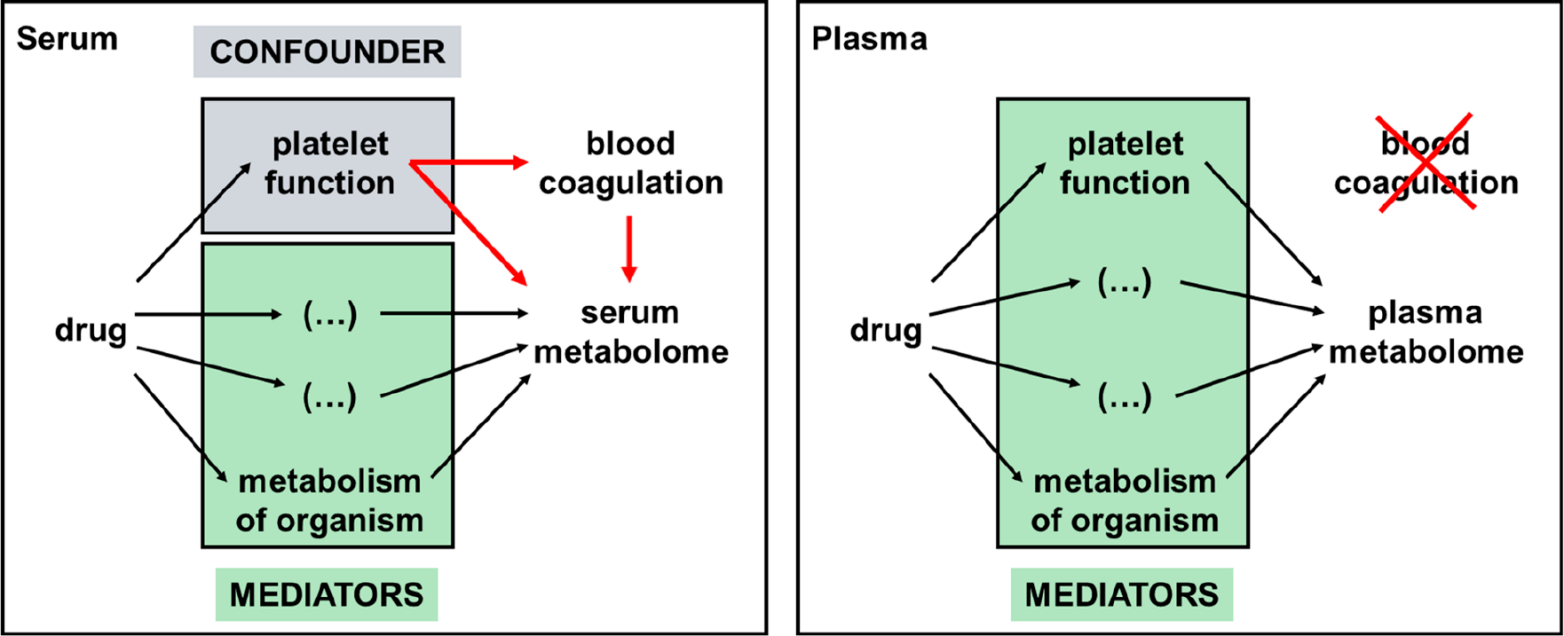
Platelets are the main confounders in serum metabolomics. The functional state of platelets *in vivo* is affecting blood coagulation during sample preparation and the serum metabolome. Thus, the functional state of platelets represents a confounder for the metabolome composition of serum. This does not apply when analyzing plasma. Here, no coagulation takes place and the functional state of platelets represents a mediator.

When drug effects on metabolism are investigated in humans, serum and plasma represent the most important sample sources. Importantly, blood coagulation specifically occurs during serum production. The functional state of platelets *in vivo* may clearly affect molecular alterations accompanying blood coagulation (Figure 6). On the other hand, the functional state of platelets may have direct effects on the serum metabolome (Figure 6). Platelets are entities with active metabolism; upon activation, they may consume, synthesize, and release various metabolites. Thus, the functional state of platelets represents a confounder for the metabolome composition of serum after coagulation (Figure 6). This does not apply when analyzing plasma, as no coagulation takes place thereby.

The present data indicated that several drug-induced changes of the serum metabolome may not mirror the patient metabolomic states but rather reflected consequences of the applied drug on platelet metabolism during blood coagulation. Thus, platelets were identified as relevant confounders for the serum metabolome but not the plasma metabolome. This finding may have important practical implications. Evidently, there is a continuous rise in the number of metabolomics papers published that are based on serum or plasma analyses. Typically, such metabolomics studies are intended to increase our current understanding of disease mechanisms and therapeutic options. However, the inherent mechanistic structure of data linking metabolic states with disease processes and understanding the consequences of drug treatment are by far not trivial. In such a complex chain of events, many important mechanisms and players may remain unobserved or unrecognized, rendering data interpretation inaccurate.

This data interpretation challenge is clearly documented in the present study. Here, we have described *in vivo* metabolomics alterations of acetylsalicylic acid affecting sphingosine-1-phosphate, 15-deoxy-PGJ2 and omega-3-fatty acid, which may help to explain known clinical effects and which may be of great relevance to better assess any potential risk associated with long-term drug treatment. As the metabolomics methodology is becoming more and more sensitive, we can also expect to observe more and more correlations between metabolic alterations and drug effects or diseases in the future. DNA-based biomarkers may be derived from specific and unique relationships between a given mutation and a disease. In contrast, disease-associated metabolic alterations are often related to indirect effects, and a specific relation of a given metabolite with a disease mechanism can hardly be expected as most metabolites may be formed and degraded by multiple chemical reactions taking place during different processes. While we have previously experienced that as little as the alteration of a single amino acid may profoundly affect relevant immune functions such as macrophage stress responses in case of glutamine,^49^ we expect that diseases or drug effects will correlate with metabolic signature profiles rather than single metabolites. Therefore, we should optimize our methodological repertoire to detect as many metabolic alterations in human individuals as possible in a reliable and unconfounded fashion.

The main limitations of this study relate to the limited sample size and the limited statistical power. Furthermore, the measurement matrix represented by serum and plasma differs slightly, potentially accounting for some differences in matrix effects. However, the events demonstrating the confounding contribution of platelets to serum metabolomics data are robust and will hardly be affected by these limitations.

## Conclusion

This study highlights the implications of potential confounders on a complex data structure typically prevalent in biomedical studies. Due to the inherent lack of robustness and complexity of metabolic profiles, a methodological standardization of metabolomics workflows is highly desirable. Here we suggest that plasma metabolomics may be better suitable for clinical studies than serum metabolomics as plasma data will not suffer from additional variance introduced by varying platelet counts as well as differing platelet states and data will not be confounded by platelet metabolism affected during blood coagulation.

## Data Availability

All proteomics data were submitted to the ProteomeXchange Consortium (http://proteomecentral.proteomexchange.org) and are available in the PRIDE partner repository^31^ with the data set identifier PXD041781 and PXD041785. Metabolomics data as well as data derived from the lipid mediator analysis are available at the NIH Common Fund’s National Metabolomics Data Repository (NMDR) Web site, the Metabolomics Workbench, https://www.metabolomicsworkbench.org,^32^ where they have been assigned to following studies: Oxylipins of serum and plasma samples: Study ID ST003050, directly accessible via its Project DOI: 10.21228/M88147. Oxylipins of platelet samples: Study ID ST003049, directly accessible via its Project DOI: 10.21228/M88147. Plasma and serum metabolomics: Study ID ST003069, directly accessible via its Project DOI: 10.21228/M88147.

## Supporting Information Available

**Supplementary Table S1**: Cohort Description (XLSX)
**Supplementary Table S2**: List of internal oxylipin standards (XLSX)
**Supplementary Table S3**: Inclusion list covering 33 *m*/*z* values specific for well-known eicosanoids and precursor molecules (XLSX)
**Supplementary Table S4**: Degree of identification of eicosanoids identified in serum/plasma as well as platelet releasates, separately in respective sheets (XLSX)
**Supplementary Table S5**: Analytical Figures of Merit regarding the MxP Quant 500 kit (XLSX)
**Supplementary Table S6**: Comparison of serum and plasma regarding proteins, eicosanoids and metabolites, separately in respective sheets; In case of proteins, log_2_-transformed label-free quantification (LFQ) values are listed for each sample (XLSX)
**Supplementary Table S7**: Comparison of activated (act) versus control (con) platelet releasates regarding proteins and eicosanoids, separately in respective sheets; In case of proteins, log_2_-transformed label-free quantification (LFQ) values are listed for each sample (XLSX)
**Supplementary Table S8**: Proteins and lipid mediators differing serum and plasma are derived from platelets; Log2 fold-changes of molecules as well as Benjamini–Hochberg adjusted *p*-values of significantly upregulated molecules in serum, when compared to plasma, are shown; For each molecule it is indicated whether it was found in the supernatant of platelets and whether it was significant upon activation (XLSX)
**Supplementary Table S9**: Acetylsalicylic acid effects in plasma and serum, respectively, regarding proteins eicosanoids and metabolites, separately in respective sheets; In case of proteins, log_2_-transformed label-free quantification (LFQ) values are listed for each sample (XLSX)
**Supplementary Table S10**: Omega3 effects in plasma and serum, respectively, regarding lipid mediators (XLSX)

## Abbreviations

11,12-DiHETrE: 11,12-dihydroxy-5*Z*,8*Z*,14*Z*-eicosatrienoic acid
17(18)-DiHETE: 17(18)-dihydroxy-eicosatetraenoic acid
5-oxo-ETE: 5-oxo-eicosatetraenoic acid
ACN: acetonitrile
CPDA: citrate-phosphate-dextrose-adenine
EPA: eicosapentaenoic acid
EtOH: ethanol
FA: formic acid
FDR: false discovery rate
HETE: hydroxyeicosatetraenoic acid
LC-MS/MS: liquid chromatography–tandem mass spectrometry
LFQ: label-free quantification
MeOH: methanol
PCA: principal component analysis
PGE2: prostaglandin E2
PRP: platelet rich plasma
SDS: sodium dodecyl sulfate
SPE: solid phase extraction
TEAB: triethylammonium bicarbonate
TGs: triglycerides
TXB2: thromboxane B2
